# Sero-survey of rubella IgM antibodies among children in Jos, Nigeria

**DOI:** 10.1186/1743-422X-8-244

**Published:** 2011-05-19

**Authors:** Surajudeen A Junaid, King J Akpan, Atanda O Olabode

**Affiliations:** 1 Department of Virology, Federal College of Veterinary and Medical Laboratory Technology, National Veterinary Research Institute, Vom- Nigeria

**Keywords:** Sero-survey, rubella IgM antibodies, children, Nigeria

## Abstract

Sero-survey of rubella IgM antibodies was carried out among children aged 0-10 years in Jos, Nigeria. Blood samples were collected from the subjects and sera extracted. Of the 93(100%) assayed for the rubella IgM antibody, 42(45.2%) were seropositive for rubella IgM antibody while 51(54.8%) were seronegative. A breakdown of the seropositive subjects reveals that 14(15.1%) of the infected children were males while 28(30.1%) were females. Those subjects within the age groups of 1-2, 3-4 and 5-6 years had the highest prevalence of 8(8.6%) followed by those within the age groups of 7-8, 9-10 years with 7(7.5%). Blood transfusion as a risk factor did not show any significant influence on the status of the subjects. The demographic data of the mothers of the subjects were also linked with the seropositivity of the children.

## Introduction

Rubella infection is caused by an RNA virus. The symptoms of rubella infection include a rash, low-grade fever, arthralgia, and lymphadenopathy. In most cases, the disease is self-limiting and rarely causes complications. Nevertheless, it causes congenital rubella syndrome (CRS) when the infection occurs during the first trimester of gestation. Complications of CRS may include miscarriage and severe abnormalities of the fetus, such as cataracts, retinopathy, heart defects, neurological deficits, and deafness. No antiviral drugs are available for treating rubella or preventing transmission to the fetus. Vaccination programs are regarded as an effective tool to eliminate rubella and congenital rubella [1-3] and [4].

As per the World Health Organization (WHO) estimate worldwide, more than 100,000 children are born with congenital rubella syndrome [5].

Rubella usually begins with malaise, low-grade fever, and a morbilliform rash appearing on the same day. The rash starts on the face, extends over the trunk and extremities, and rarely last more than 3 days. No feature of the rash is pathognomonic for rubella. Unless an epidemic occurs, the disease is difficult to diagnose clinically, as the rash caused by other viruses (e.g. enteroviruses) is similar [6] and [7]. However, the disease poses a particular threat to the developing fetus if contracted during early pregnancy. In utero, infection of the fetus may result in congenital deformity or other consequences of congenital rubella syndrome [8].

Rubella epidemics are, or have been, a world-wide phenomenon. Before the introduction of vaccine in countries such as Australia, United States of America, the United Kingdom and European Countries, rubella epidemics occurred in cycles of 6-9 year Interval [9]. In U.S.A, before the introduction of the vaccine, a single epidemic resulted in 20,000 infants being born with permanent damage due to intrauterine infection with rubella virus [10]. Elsewhere, while the Immune Status of many populations regarding rubella is less clear, some data have been reported. In Saudi Arabia, the antibody prevalence among girls aged 5-25 years has been reported to be 92% [11]. In some Africa Countries, 80% of children have been found to be positive for rubella antibodies by the age of 10 years [12]. Post-epidemic rubella antibody prevalence in Ghana has been found to be 92% among pregnant women, with susceptibility associated with a younger age [13]. In Eritrea, the prevalence of antibodies to rubella has been reported to be as high as 99% in some female population [14]. In Nigeria, rubella antibody prevalence in women of child bearing age has been reported to be 77% [15]. Some of these studies have reported an early age of exposure to rubella [12] and [13]. The highest sero-prevalence has been seen in age group as young as 5-9years and in pre-school children [16] and [17].

The general aim of this study is to determine serological evidence of recent rubella infection among children in the study area, since children could harbor the virus and serve as a good source of spread in a community, and since statistics on prevalence of rubella infection are scarce in Nigeria and particularly, the study area.

## Materials and methods

### Study area

The research was carried out in Jos City. Jos is the capital of Plateau state and is centrally situated in Plateau State, Nigeria. Jos University Teaching Hospital was chosen as Sample Collection Centre.

### Ethical consideration

The ethical clearance for this research was granted by the Jos University Teaching Hospital (JUTH) ethical Committee after due process had been followed. Before the collection of sample, information regarding the study was explained to the parents of the subjects (Children). Oral and written consent for participation in the study was obtained.

### Exclusion criteria

Only children that fall within the age range 0-10years were 	selected for this study. Those falling out of this range were excluded from this study.

### Sample collection

Samples were collected from June to October; 2007. The entire work was conducted over a 6month period.

A total of 93 blood samples were collected from children who fall within the age range of 0-10 years using sterile syringe and needles. Questionnaires were also filled by parents who accompanied the children. Between 2 to 3 millilitres (mls) of blood was collected depending on the age of the child.

### Treatment of the samples

The blood samples were allowed to clot, and then centrifuged at 3000rpm for 5 minutes. The sera were then harvested into clean sterile bottles and were then frozen at -20°c until needed for assay.

### The test

The test was carried out using enzyme linked immunosorbent assay method (ELISA). ELISA has been shown to be a sensitive and reliable procedure for detection of antibodies to rubella with diagnostic sensitivity of 98% and diagnostic specificity of ≥ 98%. The ELISA Kit used for this test was prepared and manufactured by BIOTEC laboratories Ltd, 32 Anson Road, Suffolk, UK. The test procedures were performed according to the manufacturer's instructions.

### Calculation of result

Results were calculated as indicated by the prospectus.

The results were reported as activity index values (A.I). The A.I. compares the binding activity (positivity) of the test samples to the cut-off level 0f activity that is defined as positive (Activity index value-1.0). The A.I. values of the test samples were deduced from a semi log graph of Absorbance reading of the calibrators against the assigned activity index value listed on the vials of the negative, low positive and high positive controls. Accordingly, results below 0.90 were considered negative and those above 1.10 were considered positive. Values between 0.91 and 1.09 were considered indeterminate results. There was no indeterminate result in the tested samples.

### Statistical analysis

The data obtained were analyzed with SPSS version 14.0 software program. Chi square (X^2^) was used to test the significance of variables

## Result

Table 1 shows the prevalence of rubella among children tested for rubella IgM antibody. It revealed that 42(45.2%) out of 93 children tested were positive for rubella IgM antibody whereas 51(54.8%) were negative. The distribution of the positive subjects in relation to sex shows that 14(15.1%) of the infected children were males while 28(30.1%) were females.

**Table 1:** Status of rubella in relation to sex

		Rubella status		Total	P-value
				
		Positive	Negative		
Sex	Male Count	14	37	51	
	% of total	15.1%	39.8%	54.8%	
					
	Female Count	28	14	42	0.15
	% of total	30.1%	15.1%	45.2%	
					

**Total**	Count	42	51	93	
	% of Total	45.2%	54.8%	100.0%	

A breakdown of the infected subjects with respects to their age groups is shown in table 2. It revealed that the age groups 1-2, 3-4 and 5-6 years had the highest number of infected children which is 8(8.6%) in each age group. It was followed by age groups 7-8 and 9-10 years which had 7(7.5%) of infected children in each group while age group <1 years had the least number of infected children of 4(4.3%).

**Table 2:** Prevalence of rubella IgM in relation to age group of the children

Age group of children		Rubella status		Total	P-value
				
		Positive	Negative		
< 1 year	Count	4	7	11	
	% of Total	4.3%	7.5%	11.8%	
					
1-2	Count	8	11	20	
	% of Total	8.6%	11.8%	20.4%	
					
3-4	Count	8	12	20	
	% of Total	8.6%	12.9%	21.5%	0.897
					
5-6	Count	8	8	16	
	% of Total	8.6%	8.6%	17.2%	
					
7-8	Count	7	5	12	
	% of Total	7.5%	5.4%	12.9%	
9-10	Count	7	8	15	
	% of Total	7.5%	8.6%	16.2%	

**Total**	Count	42	51	94	
	% of Total	45.2%	54.8%	100.0%	

Of the 42(100%) children infected, 42(100%), 39(92.8%) and 8(19.6%) had shown history of fever, rashes and blood transfusion respectively (Table 3).

**Table 3:** Sero prevalence in relation to history of blood transfusion, interactions with elders, fever and rashes

Variables	Frequency (%)	Rubella Status		Total
		Positive (%)	Negative (%)	
**History of blood transfusion**				

Yes	21(22.6)	8(8.6)	13(14.0)	21(22.6)
No	72(77.4)	34(36.6)	38(40.8)	72(77.4)

**Total**	93(100%)	42(45.2%)	51(54.8%)	93(100%)

**History of Interaction with elderly people**				

Yes	93(100%)	42(45.2)	51(54.8)	93(100)
No	0(0%)	0(0%)	0.(0)	0(0)

**Total**	93(100%)	42(45.2%)	51(54.8%)	93(100%)

**History of Fever**				

Yes	79(85)	39(42.0)	40(43.0)	79(85)
No	14(15)	3(3.2)	11(11.5)	14(15)

**Total**	93(100%)	42(45.2%)	51(54.8%)	93(100%)

**History of Rashes**				

Yes	72(77.4)	39(42)	33(35.4)	72(77.4)
No	21(22.6)	3(3.2)	18(19.4)	21(22.6)

**Total**	93(100%)	42(45.2)	51(54.8%)	93(100%)

Seroprevalence of rubella in the subjects, in relation to the demographic data of the mothers indicated that 25(26.9%), 13(14.0%) and 4(4.3%) represents 20-29, 30-39 and 40-49 years age group of the mothers of the rubella seropositive subjects. 20(21.5%), 12(12.9%), 8(8.6%) and 2(2.2%) of the seropositive subjects had mothers who were house wives, civil servants, business women and others respectively. The highest prevalence of rubella was observed in children whose mothers were house wives. The educational status of the mothers of the rubella seropositive children revealed that the highest prevalence occurred in children whose mothers must have had attained secondary school. Children whose mothers were married had the highest prevalence of 36(38.7%) compared to single mothers with 6(6.5%) and divorced mothers with 0(0.0%). Children whose mothers were involved in monogamy type of marriage had the highest prevalence of 23(24.7%) positivity while those whose mothers were involved in polygamy had 19(20.4%) out of the total number of children enrolled (Table 4).

**Table 4:** Sero prevalence in relation to the demographic data of the mothers

Variables (Mothers)	Rubella status of the children		Total (%)	P-value
			
	Positive no. (%)	Negative no. (%)		
**Age group in years**				

20-29	25(26.9%)	18(19.4%)	43(46.2%)	
30-39	13(14.0%)	31(33.3%)	44(47.3%)	
40-49	4(4.3)	2(2.2)	6(6.5%)	0.015

**Total**	42(45.2)	51(54.8)	93(100.0%)	

**Occupation**				

Housewife	20(21.5)	31(33.3)	51(54.8%)	
Civil Servant	12((12.9)	11(11.8)	23(24.7%)	
Business Women	8(8.6)	8(8.6)	16(17.2%)	0.594
Others	2(2.2)	1(1.1)	3(3.2%)	

**Total**	42(45.2%)	51(54.8%)	93(100.0%)	

**Education**				

Primary	15(16.1)	23(24.7)	38(40.9%)	
Secondary	19(20.4)	19(20.4)	38(40.9%)	
Tertiary	7(7.5)	8(8.6)	15(16.1%)	0.828
None	1(1.1)	1(1.1)	2(2.2%)	

**Total**	42(45.2%)	51(54.8%)	93(100.0%)	

**Marital Status**				

Single	6(6.5)	3.(3.2)	9(9.7%)	
Married	36(38.7)	47(50.5)	83(89.9%)	0.271
Divorce	0(0.0)	1(1.1)	1(1.1%)	

**Total**	42(45.2%)	51(54.8)	93(100.0%)	

**Type of Marriage**				

Polygamy	19(20.4)	25(26.9)	44(47.3%)	
Monogamy	23(24.7)	26(28.0)	49(52.7%)	0.716

**Total**	42(45.2)	51(54.8)	93(100.0%)	

## Discussion

Rubella is known to be a common cause of Maculopapular rash illness with fever and is also a childhood disease that can be either symptomatic or asymptomatic [18]. Of the 93(100%) children assayed for rubella IgM antibodies, 42(45.2%) were positive. This is a highly significant value indeed, since in United States, a single case of rubella infection is considered a potential out break [19]. It implies that the infected children have the potential to transmit the infection to others in a congregate environment like house holds, day cares, schools, places of worship and other social gathering [19]. The Medical Practitioners especially women of child bearing age who are not immuned to rubella are at risk if they come in contact with these infected children because they may contract the infection at their first trimester or second trimester and transmit it vertically to the developing fetus and hence, risk of congenital rubella syndrome [20].

Gender wise distribution of the positive subjects showed no significant difference between male and female. The same was observed in a study conducted in Chandigarh in 1874 [21] and in Bolivia and Turkey [22] and [4]. This implies that in the control of rubella, immunization should be given to all male and female children to reduce the circulation of the virus in a community [23].

This study observed a slight increase in seropositivity of 8(8.6%) in children that fall within the age groups 1-2, 3-4 and 5-6 years. The slight increase of positivity in these age groups may be due to risk factors like frequent exposures to already infected people in congregate environments like schools and play grounds [19].

Of the 42 (100%) positive cases, 39(92.8%) showed classical history of rashes which is in accord with the fact that some people could be asymptomatically or symptomatically infected with rubella [18].

The demographic data (Age, occupation, educational status, marital status and type of marriage) of the mothers of these children were also linked with the rubella positivity and negativity of these children. The rubella IgM positivity of the children assayed decreases with an increase in the age of their mothers. This could be attributed to the fact that mothers of younger ages visit congregate environment frequently with their children and thereby exposing their children to several risk of contracting rubella via contact or respiratory route. Children whose mothers were full time house wives had the highest prevalence of 20(21.5%) unlike civil servant 12(12.9), business women 8(8.6%) and others 2(2.2%). Children whose mothers must have had attained secondary schools were the mostly infected with 19(20.4%) positivity, unlike primary 15(16.1%), tertiary 7(7.5%) and none 1(1.1%). Marital status of the mothers also influenced the status of rubella among the studied group. Children whose mothers were married were the ones most infected. The possible reason may be as a result of high level of contact/Interactions at the family level which promotes rubella transmission since rubella Seroprevalence increases as the number of sibling's increases [24].

## Conclusion

Rubella infection control is essential for eliminating indigenous and preventing CRS. The strategies for rubella outbreak control include defining target population for rubella vaccination, ensuring that susceptible persons within the target populations are vaccinated rapidly and maintaining rubella and CRS surveillance. Control measures should be implemented as soon as a case of rubella is identified. Maintaining control measures is essential when pregnant women are possible contacts of patients with rubella. Susceptible pregnant women who are exposed to rubella should be thoroughly evaluated for possible rubella infection.

## Recommendation

It is recommended that a policy of mass vaccination of children should be put in place in order to effectively control rubella which could be circulated to women of child bearing age who are the ones most at risk of contracting rubella infection.

## Competing interests

The authors declare that they have no competing interests.

## Authors' contributions

SAJ and KJA participated in the design of the study and performed the investigation, analysis and interpretation of data. AOO participated in the design and coordination. SAJ and KJA drafted the manuscript while SAJ also working as the corresponding author. All authors read and approved the final manuscript.

## References

[B1] Ching-Chiang L Chun-Yuh Y Ching-Tang S Bai-Hsiun C Yeou-Lih H Rubella Seroepidemiology and Catch-up Immunization among Pregnant Women in Taiwan: Comparison between Women Born in Taiwan and Immigrants from Six Countries in Asia Am J Trop Med Hyg 2010 82 1 40 44 10.4269/ajtmh.2010.09-0302 20064993 PMC2803507

[B2] Dontigny L Arsenault MY Martel MJ Biringer A Cormier J Delaney M Gleason T Leduc D Martel MJ Penava D Polsky J Roggensack A Rowntree C Wilson AK Society of Obstetricians and Gyneacologist of Canada, Rubella in pregnancy J Obstet Gynaecol Can 2008 30152 158 [PubMed] 10.1016/S1701-2163(16)32740-2 18254998

[B3] Best JM Rubella Semin Fetal Neonatal Med 2007 12 182 192 [PubMed] 10.1016/j.siny.2007.01.017 17337363

[B4] Kanbur NO Derman O Kutluk T Kinik E Age specific Rubella Sero prevalence of an Unvaccinated population of Adolescents in Ankara, Turkey Jpn J Infect Dis 2003 56 23 25 12711822

[B5] Vijayalakshmi P Anuradha R Prakash K Narendran K Ravindran M Rubella Sero-surveys at three Aravid Eye Hospitals in Tamil Nadu, India Bull World Health Organization 2004 82 259 264 PMC2585953 15259254

[B6] Wolinsky JS Fields, BN; Knipe, DM Rubella Fields'Virology 1996 Philadelphia, LippenCott-Raven 899 92921598407

[B7] Geo FB Janet SB Stephen AM Jawetz, Mernick, and Adelberg's Medical Microbiology Rubella 2004 23 International edition, McGraw-Hill Companies, Asia 566 568

[B8] Cooper AR Rubella and other Exanthemas Medicine International 1988 53 2182 5

[B9] Assaad F Ljungars-Estevez K Rubella-World impact Review of Infectious diseases 1985 7 29 36 10.1093/clinids/7.Supplement_1.S29 4001731

[B10] Cochi SL Congenital rubella syndrome in the United States, 1970-1985. On the Verge of elimination American Journal of Epidemiology 1989 129 349 61 2912045 10.1093/oxfordjournals.aje.a115138

[B11] EL-Mekki AA Zaki ZM Screening of rubella antibodies among Saudi women of child bearing age Saudi Medical Journal 1998 19 575 7

[B12] Gomwalk NE Ahmed AA Prevalence of rubella antibodies in the African continent Reviews of infectious disease 1989 11 116 21 10.1093/clinids/11.1.116 2783785

[B13] Lawn JE Unseen Blindness, Unheard deafness, and Unrecorded death and disability: Congenital rubella in Kumasi, Ghana American Journal of public health 2000 90 1555 61 10.2105/AJPH.90.10.1555 11029988 PMC1446363

[B14] Sallam TA Raja'a YA Benbrake MS Alshaibani KS Al-Habani AA Prevalence of Rubella Antibodies among school girls in Sana'a, Republic of Yemen Eastern Mediterranean Health Journal 2003 12 1 4 15562744

[B15] Onyenekwe CS Okar TS Anriola OG Prevalence of rubella IgM antibodies in Women of child-bearing age in Lagos West African Journal of Medicine 2000 19 1 23 26 10821082

[B16] Clarke WL Epidemiological studies of rubella virus in a tropical African Community Bulletin of the World Health Organization 1980 58 931 5 6971191 PMC2396008

[B17] Mingle JA Frequency of Rubella antibodies in the population of some African Countries Rev Infect Diseases 1985 7 S68 71 10.1093/clinids/7.Supplement_1.S68 4001737

[B18] Gurgose MK Yilmaz E Godekmerdan A Akoa Z Dogam Y Akarsus S Aygun AD Seroprevalence of Mumps, Varicella and Rubella Antibodies in Children 1-16 years of age in eastern Turkey Turk J Pediatr 2006 48 185 188 17172059

[B19] CDC Control and Prevention of Rubella: Evaluation and Management of suspected outbreaks, Rubella in pregnant women, and surveillance for congenital Rubella syndrome MMWR 2001 50 1 23 11475328

[B20] Gregg NM Congenital Cataract following German Measles in the Mother Transactions of the Ophthalmological Society of Australia 1941 3 35 6

[B21] Pal SR Chitkara NL Broor S Murthy JG Choudhary S Davi PK Serological Investigation of Rubella Virus Infection in and around Chandigarha Preliminary Communication Indian Journal Med Res 1974 62 240 5 4442966

[B22] Bartoloni A Bastalesi F Roselli M Mantell A Dini F Carballo ES Seroprevalence of Varicella Zooster and Rubella antibodies among rural populations of the Chaco region, South-Eastern Bolivia Trop Med Int Health 2002 7 513 7 10.1046/j.1365-3156.2002.00852.x 12031073

[B23] Nalini R Murugan S Raja D Varalasmi E Ohanagara D Sero Survey of Rubella in Five blocks of Tamil Nadu Indian J Med Res 2006 123 51 54 16567868

[B24] Arroyo M Alia JM Mateos ML Carrasco JL Ballesteros F Lardinois R Natural Immunity to Measles, Rubella and Mumps among Spanish Children in the Prevaccination era Int J Epidermiol 1986 15 95 100 10.1093/ije/15.1.95 3957548

